# Robust reconstruction of time-resolved diffraction from ultrafast streak cameras

**DOI:** 10.1063/1.4985059

**Published:** 2017-06-02

**Authors:** Daniel S. Badali, R. J. Dwayne Miller

**Affiliations:** 1 Hamburg Centre for Ultrafast Imaging, Department of Physics, Max Planck Institute for the Structure and Dynamics of Matter, University of Hamburg, Hamburg 22761, Germany; 2 Departments of Chemistry and Physics, University of Toronto, Toronto, Ontario M5S 1H6, Canada

## Abstract

In conjunction with ultrafast diffraction, streak cameras offer an unprecedented opportunity for recording an entire molecular movie with a single probe pulse. This is an attractive alternative to conventional pump-probe experiments and opens the door to studying irreversible dynamics. However, due to the “smearing” of the diffraction pattern across the detector, the streaking technique has thus far been limited to simple mono-crystalline samples and extreme care has been taken to avoid overlapping diffraction spots. In this article, this limitation is addressed by developing a general theory of streaking of time-dependent diffraction patterns. Understanding the underlying physics of this process leads to the development of an algorithm based on Bayesian analysis to reconstruct the time evolution of the two-dimensional diffraction pattern from a single streaked image. It is demonstrated that this approach works on diffraction peaks that overlap when streaked, which not only removes the necessity of carefully choosing the streaking direction but also extends the streaking technique to be able to study polycrystalline samples and materials with complex crystalline structures. Furthermore, it is shown that the conventional analysis of streaked diffraction can lead to erroneous interpretations of the data.

## I. INTRODUCTION

Watching atoms move in real time may have once been a distant dream, but recent technological advancements have made it a daily occurrence in laboratories around the world. The primary obstacle in reaching this goal has been developing a probe capable of accessing the extreme spatial (sub-ångström) and temporal (femtosecond) scales associated with atomic motions. Both these requirements have been achieved with ultrashort x-ray and electron pulses (see, for instance, Refs. 1–11), and time-resolved diffraction with such probes has cemented itself as an indispensable tool for making molecular movies.

Conventionally, such experiments are performed in a pump-probe fashion, where the dynamics are initiated by pumping the sample with a femtosecond laser pulse and probing it at a later time using ultrafast x-ray or electron diffraction. Varying the delay between the arrival of the pump and the probe allows the temporal dynamics to be sampled at distinct time points during the dynamics. A complete “movie” is then made by combining the diffraction patterns at each time delay. Unfortunately, this stroboscopic procedure can require hundreds of time delays to sufficiently resolve typical structural dynamics. The limited amount of sample and degree of sampling these time points for adequate signal-to-noise severely limit the ease of performing such experiments.

An alternative mode of operation is to stretch the probe pulse to the order of a few picoseconds and then detect the resulting diffraction pattern using an ultrafast streak camera. The long probe pulse captures the transient dynamics of the sample as it diffracts, and the streak camera uses a rapidly oscillating or ramped electric field to map the temporal coordinate of the diffraction pattern to a spatial coordinate on the detector. The resulting streaked diffraction pattern then contains the temporal dynamics along one of its spatial dimensions. This offers the unprecedented opportunity to record an entire molecular movie with a single probe pulse. Ultrafast streaking is an established technique in time-resolved x-ray diffraction^12–15^ and has recently been applied to ultrafast electron diffraction.^16,17^ Modern advances in x-ray^18–24^ and electron^25–30,51^ streak cameras have reported a temporal resolution in the femtosecond regime, making streaking an attractive alternative to the traditional stroboscopic method of time-resolved diffraction.

While this technique has many challenges, one of the hindering restrictions is the lack of a rigorous, quantitative analysis of the streaked diffraction patterns. All previous studies^12–15,17^ have adopted the same approach: for each streaked diffraction spot, the image is averaged over the transverse coordinate to the streaking direction to obtain a one-dimensional trajectory of the intensity. While this is the most straight-forward approach, it obviously constitutes a loss of information; instead of a two-dimensional diffraction image at each time point, all that remains is one-dimensional data. A slight improvement was made by segmenting the streaked diffraction pattern into boxes along the streaking direction and then fitting to obtain the characteristics of each box.^16^ However, both these methods suffer from the same fundamental flaw; they subtly assume that the intensity along the streak of a diffraction spot is equal to the time-dependent intensity of that spot. This can lead to erroneous results when used to analyze even the simplest of dynamics.

In this article, this problem will be addressed by the development of a general theory of streaking of time-dependent diffraction patterns. The presented analysis technique provides a way to recover the two-dimensional diffraction image at each time point from the streaked image alone. In addition, it is demonstrated that the proposed approach works on diffraction peaks that overlap when streaked, which not only removes the necessity of carefully choosing the streaking direction but also extends the streaking technique to study polycrystalline samples and materials with complex crystalline structures, such as organic and biological crystals.

## II. GENERAL STREAKING THEORY

The goal of this work is to outline a method to recover the time-dependent diffraction pattern *u*(*x*, *y*; *t*) from a streaked image *s*(*x*, *y*). Here, *x* and *y* are the detector coordinates and *t* is the time-dependence due to the sample dynamics. The relationship between *s* and *u* can be arrived at by considering the physics behind the streaking process. Probe electrons (or electrons produced by a photocathode from probe x-rays) are given a transverse momentum kick so that electrons arriving at the sample at different times are deflected to different spatial locations on the detector. This causes the diffraction pattern to be “smeared” along the streaking direction. The streaked diffraction pattern can thus be pictured as being formed by overlapping the instantaneous diffraction patterns recorded by each temporal slice of the electron pulse. Mathematically, this means that for each time *t*, the instantaneous diffraction pattern *u*(*x*, *y*; *t*) is shifted spatially by the appropriate displacement *v_s_t*, where *v_s_* is known as the streaking velocity or the sweep speed.^31^ Of course, some parts of the electron pulses will contain more electrons than others, and so, each instantaneous diffraction pattern must be weighted by the appropriate temporal electron density *ρ*(*t*). The contribution from each temporal slice is then integrated at the detector, resulting in the streaked diffraction pattern

$$s(x,y)=\int_{-\infty }^{\infty }\rho (t)u(x-v_{s}t,y;t)\;dt+n(x,y),$$ (1)

where *n*(*x*, *y*) incorporates any measurement noise and will be assumed to be a Gaussian random variable. A more physically realistic model would incorporate Poisson noise, but this is a reasonable approximation given the relatively high signal-to-noise ratio (SNR) in typical ultrafast diffraction experiments. Equation (1) is the governing equation for the analysis of streaked, time-dependent diffraction data.

The first term on the right-hand side of Eq. (1) is sometimes known as *spatially-varying convolution*, due to its formal similarity to the conventional convolution operator. In fact, if the diffraction pattern is not changing, then *s* = *ρ* * *u* +* n*, where “*” denotes the convolution operator. This simple relationship can be used to recover *ρ*(*t*) in a separate experiment by streaking the direct probe beam or a static (un-pumped) diffraction pattern.^26^

**FIG. 1.**
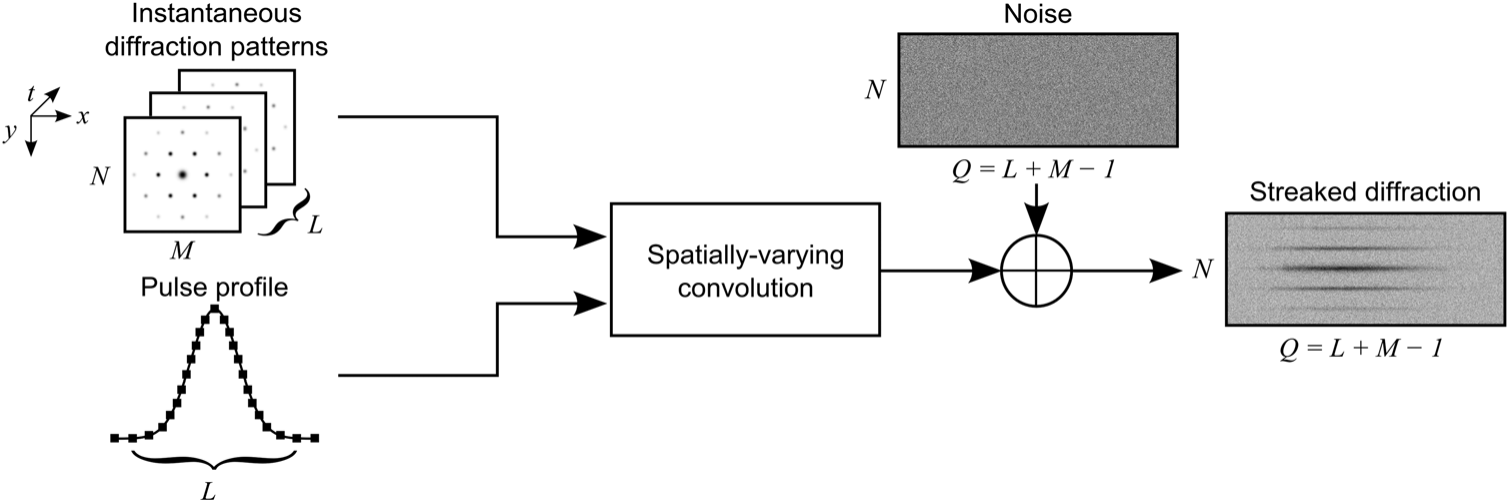
Block schematic of the linear space-variant image degradation model, with the image dimensions. The probe pulse records a stack of instantaneous diffraction patterns as it propagates through the sample, which is converted into a streaked diffraction pattern by the spatially varying convolution model, which accounts for the profile of the pulse. Finally, the noise is treated as an additive, resulting in the measured streaked diffraction pattern.

Due to the discrete nature of detection, a streaking experiment actually measures the discrete version of Eq. (1),

$$s_{i,j}=\sum_{k=0}^{L-1}\rho_{k}u_{i,j-k,k}+n_{i,j},$$ (2)

where *L* is the length of the vector ρ representing the electron pulse profile. The temporally evolving diffraction pattern takes the form of a stack of images with elements *u_i_*_,__*j*__,__*k*_. The discretization of the time coordinate of u occurs by sampling the third dimension of *u*(*x*, *y*; *t*) at the pixel locations along the *x*-axis due to the streaking. This linear model is presented in Fig. 1. The pertinent image dimensions are defined as follows:

• $\rho \in {\mathbb{R}}^{L\times 1}$,
• $u\in {\mathbb{R}}^{N\times M\times L}$,
• $s\in {\mathbb{R}}^{N\times Q}$, where *Q* = *L* + *M* − 1,
• $n\in {\mathbb{R}}^{N\times Q}$.

In writing Eq. (2), it has been implicitly assumed that the streaking direction (specified by the *x* axis) lies along a detector pixel axis. This is, however, difficult to achieve in practice, and so, all the images must be re-sampled on the grid defined by the streaking axis (see Sec. IV A). Also, because the streaking is solely along the *x*-axis by convention, each row of the streaked image can be considered independently. That is, Eq. (2) can be written as *N* independent equations of the form

$$s_{j}=\sum_{k=0}^{L-1}\rho_{k}u_{j-k,k}+n_{j}.$$ (3)

This dramatically decreases the memory requirements of the algorithm presented below, as well as enabling parallel implementations. For the remainder of this article, the *i* (or *y*) dependence will be dropped and all equations and quantities will refer to a single pixel row.

Attempting to recover u from Eq. (3) requires the computation of an *M* × 1 diffraction pattern at each of the *L* pixels along the streaking axis. To make this problem computationally tractable, we instead only solve for u at every *W*/*L* pixel, where *W* ≤* L*. The instantaneous diffraction patterns at the intermediate pixels can be computed by interpolation. The benefit of this approach is that instead of having to solve Eq. (3) for the *M* ×* L* values of *u_j_*_,__*k*_, we only need to solve for the *M* ×* W* values of a vector $U\in {\mathbb{R}}^{MW}$ formed by stacking the *W* target diffraction patterns. This construction allows Eq. (3) to be rewritten as

s=AU+n, (4)

where the matrix $A\in {\mathbb{R}}^{Q\times MW}$ is constructed from both the electron pulse profile and the interpolants and can be efficiently calculated in the Fourier domain. The procedure leading to Eq. (4) is discussed in detail in the Appendix.

This construction is extremely expressive;^32^ when *W* <* L*, this approximation consists of sub-sampling the temporal dynamics and interpolating between these discrete values. However, when *W* =* L*, the interpolants can be set to delta functions and the full temporal dynamics are recovered at each pixel along the streaking axis. Thus, the choice of *W*/*L* can be used to tune the degree of approximation.

## III. SPATIALLY VARYING DECONVOLUTION

Now armed with an understanding of the underlying physics behind the streaking process, an appropriate method to analyze such experiments can be developed. The problem at hand can be encapsulated as follows: given a streaked image s and the matrix A (which has been constructed from the measured electron pulse profile), how can the time-dependent, unstreaked diffraction pattern U be recovered from Eq. (4)? This is an inverse problem that is ubiquitous in imaging applications and is unfortunately ill-posed.^33^ Although there are several possible ways to tackle this problem, a model free approach will be adopted to limit the possibility of biasing the reconstruction.^34^ The best estimate of the time-dependent diffraction pattern U will be chosen as the value $\hat{U}$ with the highest probability given the observed streaked diffraction image s (that is, the *maximum a posteriori* estimate). Appealing to Bayes' theorem,^35^ this means solving

$$\hat{U}(\mu )=\underset{U⪰0}{\text{arg}\;\text{min}}\left\{{\left\|s-\text{AU}\right\|}_{2}^{2}+\mu \Omega (U)\right\},$$ (5)

where ⪰ denotes the element-wise non-negativity. This constraint arises from the fact that the diffraction intensity must be positive and serves to encourage physically realistic solutions. The best estimate of the time-dependent diffraction pattern U is thus chosen as the familiar least-squares solution, modified by a regularizer Ω(U) which penalizes solutions that are physically improbable. The parameter *μ* ≥ 0 allows for tuning the degree of regularization by balancing the fidelity to the data with satisfying the regularizer. A common choice for the regularization term is $\Omega (U)={\left\|\Gamma U\right\|}_{2}^{2}$, where Γ is a matrix, which is known as Tikhonov regularization.^36^ There are many potential avenues for choosing Γ to pursue based on the physical properties of *u*(*x*, *y*; *t*), such as its temporal and spatial smoothness or the symmetry of the diffraction pattern.

## IV. PRACTICAL ASPECTS OF THE RECONSTRUCTION

There are a number of practical aspects that are important when implementing a reconstruction scheme using Eq. (5). Each of these will be discussed individually below.

### A. Identification of the streaking direction

As mentioned previously, the angle between the streaking direction and the pixel axes needs to be accounted for. One possible way to identify this angle is by exploiting the mathematical transform known as the Radon transform.^37^ The Radon transform of an image is defined as the line integral of the image along a straight line at an offset *ρ* from the origin, whose normal vector makes an angle *θ* with the *x*-axis. Mathematically, the Radon transform ℝ(θ,ρ) of a streaked image *s*(*x*, *y*) is given by

$$\mathbb{R}(\theta ,\rho )\left[s(x,y)\right]=\int_{-\infty }^{\infty }\int_{-\infty }^{\infty }s(x,y)\delta (\rho -x\;\text{cos}\;\theta -y\;\text{sin}\;\theta )\;dx\;dy,$$ (6)

where *δ*(⋅) is the Dirac delta function. The mental picture of the Radon transform is the complete set of projections of the image at each angle *θ*. Intuitively, the value of the Radon transform will be larger at angles and offsets where the image looks more like a line. Since the streaked image looks like a set of parallel lines, to find the angle of a streaked diffraction image, we can take the angle that maximizes the integrated Radon transform

$$\theta_{\text{streak}}=\underset{\theta }{\text{arg}\;\text{max}}\left\{\;\mathbb{R}(\theta ,\rho )\left[s(x,y)\right]\;\right\}.$$ (7)

Note that the maximization ignores the offsets because we are only concerned with identifying the angle at which the lines appear in the image. Of course in practice, a discrete approximation of the Radon transform must be used, such as that provided by the Matlab function radon.

Once the streaking angle has been found, the streaked diffraction image should be rotated about its origin so that the streaking direction aligns with the pixel axes. There are a number of sophisticated ways to do this (see, for example, Ref. 38), and an in-depth discussion of such methods is beyond the scope of this article. In our results, we used the Matlab imrotate function.

**FIG. 2.**
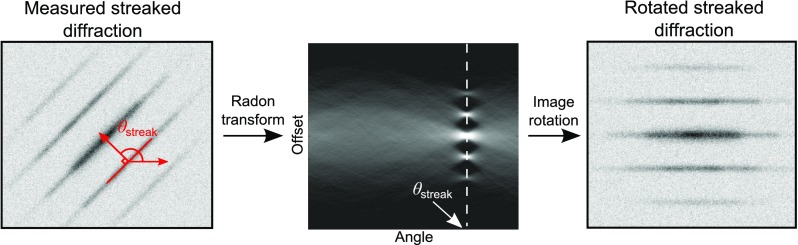
Illustration of the procedure for identifying the angle of a streaked diffraction pattern relative to the detector pixel axes. The streak angle is defined as the angle between the horizontal detector pixel axis and the normal vector to the streak lines. A Radon transform is used to convert the streaked data into projections at different rotation angles of the image. The angle at which the Radon transform achieves its maximum is the streak angle, as shown in the middle image. Finally, an image rotation algorithm can be used to align the streaked data with the detector pixel axes.

The procedure for identifying the streaking angle and rotating the streaked image is illustrated in Fig. 2.

### B. Initial guess

Key to a faithful recovery of the underlying dynamics using Eq. (5) is a good initial guess for the instantaneous diffraction patterns. Fortunately, in the majority of experiments, the structural dynamics of interest give rise to relatively small changes in the diffraction patterns. As such, a good initial guess for the instantaneous diffraction patterns is the static diffraction pattern weighted by the intensity changes observed in the streaked diffraction image. In situations in which the structural dynamics give rises to changes in the peak positions or peak widths, the streaked diffraction image can similarly be used to make an educated guess out of the static diffraction pattern.

Note that if rotation is applied to align the streak direction with the detector pixels (as discussed in Sec. IV A), then the same rotation must be applied to the static diffraction pattern when used as an initial guess.

### C. Choice of the regularization parameter

One of the most important aspects of solving Eq. (5) is the choice of the regularization parameter *μ* since it controls the balance between matching the experimental data and satisfying the regularizer. It must be carefully chosen to ensure a physically relevant solution without over-constraining the data. Fortunately, diffraction experiments have access to an extra piece of information that can inform choosing *μ*: an estimate of the amount of noise in the images. This suggests using the so-called *discrepancy principle* to choose the regularization parameter,^39^ which makes use of the fact that the measurement is contaminated by an amount ${\left\|s-\text{AU}\right\|}_{2}={\left\|n\right\|}_{2}≃\delta$, where *δ* is an estimation of the measurement noise level. The discrepancy principle observes that the reconstructed solution cannot result in less residual error than the noise in the measurement; otherwise, we would be fitting the solution to the noise. That is, the reconstruction is acceptable if

$${\left\|s-A\hat{U}(\mu )\right\|}_{2}\leq \delta .$$ (8)

Thus, because the experimental data can differ from the “true” streaked diffraction pattern by an amount *δ*, the regularization parameter should be chosen as the largest value that allows the reconstructed streaked image to remain within this noise level. This provides the maximum allowed regularization while still remaining faithful to the experimental data. Practically, the discrepancy principle is implemented as choosing *μ* as the zero of the function

$$f(\mu )={\left\|s-A\hat{U}(\mu )\right\|}_{2}^{2}-c^{2}\delta^{2},$$ (9)

where *c* ≥ 1 is a small constant (*c* = 1.1 is typical). In many cases, Eqs. (9) and (5) must be solved iteratively; although if Tikhonov regularization is used and Γ is invertible, then *μ* can be computed from Eq. (9) without knowledge of $\hat{U}$ through the use of singular value decomposition.^40^

Mathematically, the noise parameter *δ* is defined by $\delta^{2}=E\left({\left\|n\right\|}_{2}^{2}\right)$, where E( · ) represents the expectation value. To devise a scheme to measure this quantity experimentally, we first note that each element of the vector n is a zero-mean normally distributed random variable with a standard deviation of *σ* (this follows from the assumption that the noise is Gaussian). Using the laws of probability, we thus know that the quantity ${\left\|n\right\|}_{2}^{2}$ is distributed as *σ*^2^*χ*^2^(*Q*), where *χ*^2^(*Q*) is a chi-squared distribution with *Q* degrees of freedom. Since the expectation value of this distribution is *Qσ*^2^, we arrive at the relation

$$\delta =\sqrt{Q}\sigma .$$ (10)

Thus to measure *δ* experimentally, one only needs to know the dimension of the streaked image along the streaking direction and to measure *σ*. The latter can be measured by finding a region of the streaked diffraction image without any signal and calculating the standard deviation of the pixel values in that region.

### D. Complete algorithm

To summarize the theory and practical details that have been presented thus far, Algorithm 1 outlines the sequence of steps required to analyze streaked ultrafast diffraction:

**Algorithm 1:** Spatially varying deconvolution

1: Measure streak of the electron pulse without a sample
2: Calculate ρ
3: Measure streaked diffraction with excitation
4: Measure noise level *δ*
5: Choose interpolation kernels
6: Construct A from ρ and the interpolants as outlined in the Appendix
7: Rotate the streaked image to align with detector pixel axes
8: **for** each row in the streaked image **do**
9: Calculate initial guess
10: Calculate regularization parameter *μ* from Eq. (9)
11: Calculate $\hat{U}$ from Eq. (5)
12: Interpolate to get the time-dependence of the row of the instantaneous diffraction u
13: **end for**

## V. RESULTS

To demonstrate the versatility and flexibility of this procedure, a wide range of temporal dynamics were simulated to cover the most common behavior of transient diffraction patterns. In general, changes in the intensities, positions, and widths of the diffraction peaks account for the full range of possible changes that can arise from a laser-irradiated crystal. For instance, changes in peak intensities could be attributed to the temperature increase due to the Debye-Waller effect, changes in peak positions are related to expansions/contraction of the unit cells, and changes in the peak widths could result from the presence of acoustic phonons.

The spatially varying deconvolution approach, which culminates in Eq. (5) and Algorithm 1, was applied to simulated streaked time-dependent diffraction patterns. Each diffraction pattern was an *N* ×* M* = 32 × 32 image streaked over *L* = 256 pixels by a temporally Gaussian pulse profile ρ with a standard deviation of *L*/2. The initialization of the dynamics (i.e., no delay between the pump and the probe) occurred at pixel number *L*/2. Poisson noise was added to the images at a signal-to-noise ratio (SNR) of 50, unless otherwise stated.

Minimization of Eq. (5) was performed using a simple steepest descent algorithm with the step size chosen according to a backtracking line search as implemented in Ref. 41. The positivity of U was enforced by a two-point bound projection method. The interpolants were chosen to be 50% overlapping symmetric Hann windows, and *W* =* L*/8 interpolants were used. The initial guess for the instantaneous diffraction patterns was chosen as the static diffraction pattern weighed by the intensity of the streaked pattern at each of the *W* sampling points. All computations were performed using custom-written software in MATLAB R2015a (The Mathworks, USA).

**FIG. 3.**
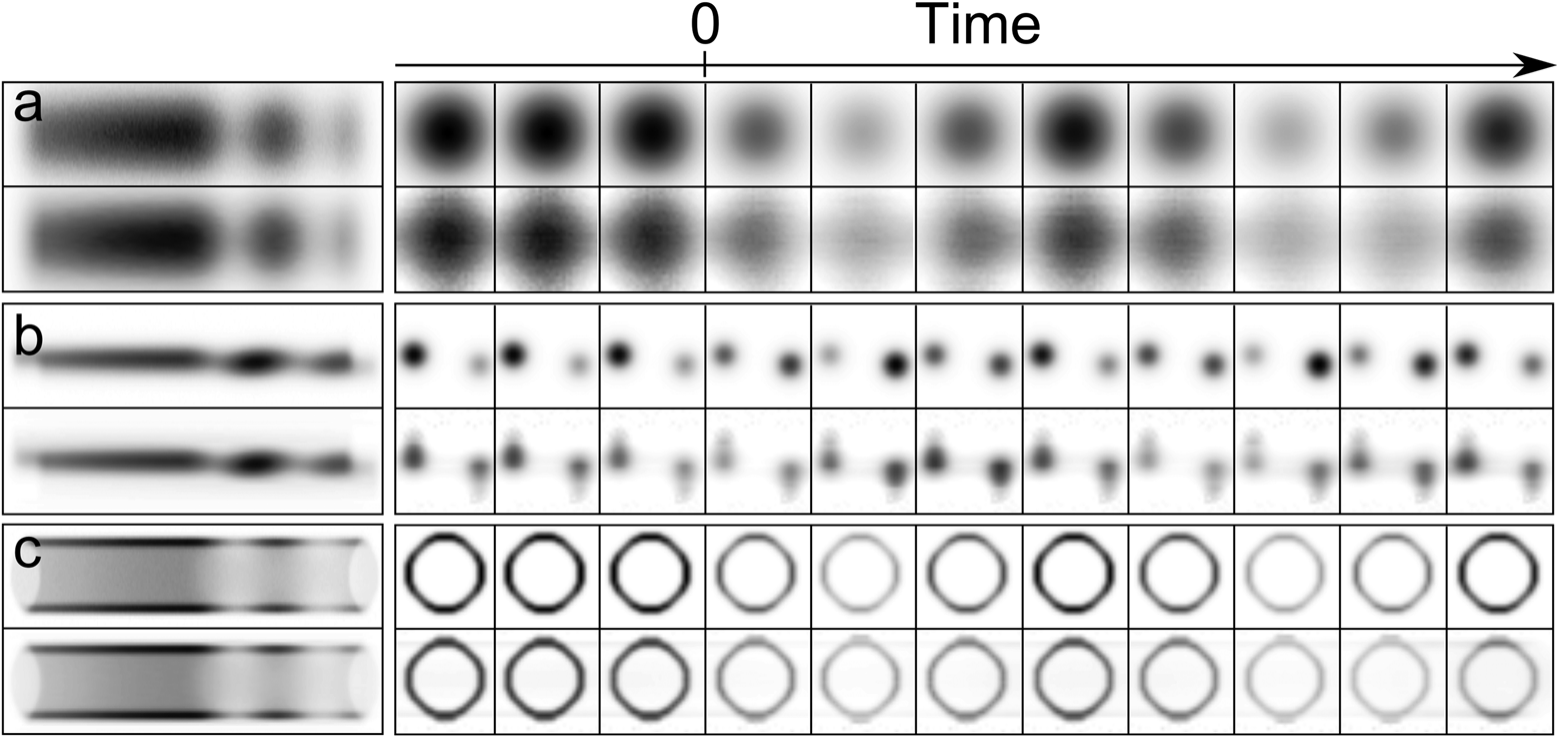
Demonstration of the spatially varying deconvolution algorithm's ability to recover time-dependent diffraction patterns from simulated streaked diffraction data. Amplitude changes for various types of diffraction patterns are shown: (a) a single diffraction spot, (b) two overlapping spots, and (c) a diffraction ring. The upper row of each panel contains selected “true” (input) instantaneous diffraction patterns, and the lower rows show the corresponding recovered (output) patterns. Each row is shown with the same grayscale. The leftmost images show the simulated and recovered streaked data.

Figure 3 summarizes the results on a variety of simulated data. Each of the three rows explores conditions that can occur for samples of differing crystalline qualities: (a) an isolated diffraction peak that could be due to a simple crystal, such as a metal, (b) overlapping diffraction peaks that would be difficult to avoid for complex crystals such as organic or biological samples, and (c) a diffraction ring that would occur for polycrystalline samples. The top panel for each dynamic is simulated data that would be measured in a streaking experiment (left column) versus a conventional stroboscopic experiment (right column), with the corresponding images recovered by the algorithm shown below.

From visually inspecting these images, it is evident that there is a qualitative agreement between the input and recovered diffraction patterns. Comparing the two sets of images confirms that the shape, position, and intensity of the diffraction pattern are fairly accurately reproduced, perhaps with a few small artifacts. Similar quality results were obtained for changes in the position and shape of a single diffraction spot (data not shown). Furthermore, the quality of the reconstruction suggests that the assumption of Gaussian noise as opposed to Poisson noise is acceptable.

A Tikhonov matrix Γ was constructed to enforce temporal smoothness. In all cases, the regularization parameter computed by the discrepancy principle was very small, e.g., *μ* ≈ 0.001 or smaller, which indicates that very little regularization was required to obtain an acceptable reconstruction for the simple simulated data considered here. As such, no regularization was used for the results presented in Fig. 3.

The middle panel of Fig. 3 contains two small diffraction peaks with overlapping streaked trajectories that oscillate in intensity. In all previous streaking experiments, this is a situation that has been carefully avoided since the traditional analysis technique makes it impossible to separate the dynamics of the two spots individually. However, as ultrafast diffraction is being applied to samples with more complicated crystal structures, overlapping diffraction spots might be unavoidable in future streaking experiments. Because of this, it is interesting to see how the spatially varying deconvolution of this work performs in such cases. The recovered instantaneous diffraction patterns in Fig. 3(b) indicate that Algorithm 1 is powerful enough to consider overlapping diffraction spots since the presence of two diffraction spots is successfully reproduced.

To further explore the capacity of this technique, Fig. 3(c) considers a diffraction pattern composed of a ring. Again, the instantaneous diffraction patterns were successfully recovered from a single streaked image. These two lower panels demonstrate that using the appropriate analysis opens up streaked ultrafast diffraction to a broad class of materials, such as polycrystalline, amorphous, and organic samples. These are categories of materials that were thought to be inaccessible to the streaking technique and so have gone unexplored in the past.

**FIG. 4.**
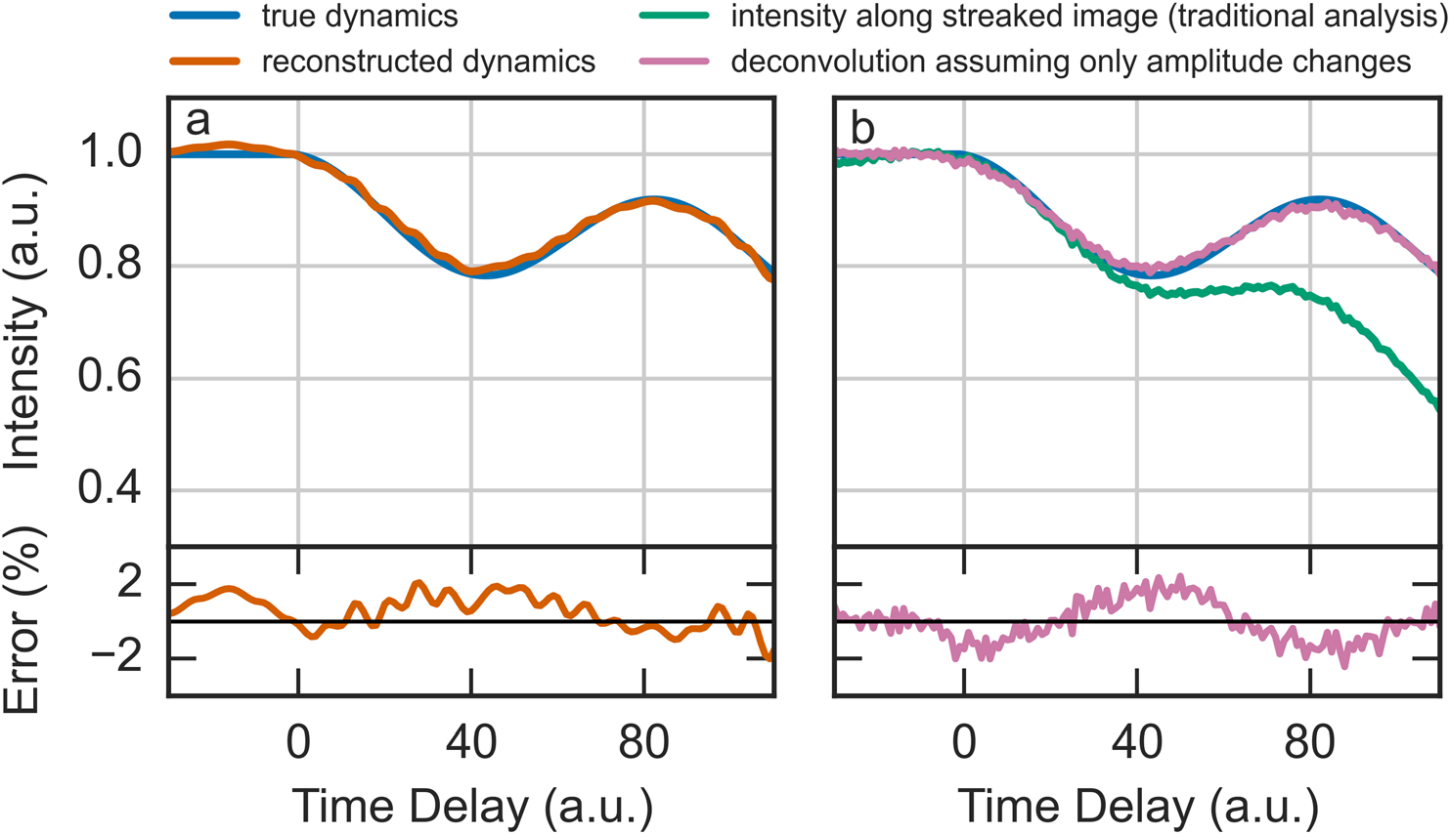
(a) Comparison of the time-dependent intensity of the recovered diffraction spot shown in Fig. 3(a) (orange). Shown for reference is the true amplitude that was used to generate the data (blue). (b) The intensity recovered by the traditional analysis method (green) and the simplified deconvolution approach derived in Eq. (11). Shown in the bottom panels are the residual percent errors between the recovered and true intensities.

To quantify the fidelity of the reconstruction, Fig. 4 plots the average diffraction spot intensity as a function of the time delay taken from the sequence of images in Fig. 3(a). Figure 4(a) compares the input “true” intensity that was used to simulate the data with the intensity recovered by Algorithm 1. The bottom panel shows the residual error, defined as the relative difference between the recovered and true intensity, which has a standard deviation of 0.9%. In contrast, Fig. 4(b) shows the result obtained by the traditional approach of taking the intensity profile along the streaked image. It is observed that the traditional method both severely underestimates the amplitude and the period of the oscillations and overestimates the decay time of the dynamics. This is because the intensity from the traditional approach is significantly impacted by the shape of the pulse profile, which decays at larger time delays. Fortunately, because the dynamics in Fig. 3(a) consisted of only an amplitude change, Eq. (1) gives rise to a simpler analysis. If the instantaneous diffraction pattern is factored as *u*(*x*, *y*; *t*) = *a*(*t*)*u*_0_(*x*, *y*), where *a*(*t*) is the time-dependent amplitude and *u*_0_(*x*, *y*) is the spatial diffraction pattern, Eq. (1) simplifies to

$$s(x,y)=\int_{-\infty }^{\infty }\rho (t)a(t)u_{0}(x-v_{s}t,y)\;dt+n(x,y)=\left[a\rho \right]*u_{0}+n.$$ (11)

In this case, [*a*(*t*)*ρ*(*t*)] can be recovered by using a traditional noisy deconvolution technique such as Wiener deconvolution to remove the influence of the spot profile. The amplitude dynamics can then be calculated by dividing by the pulse profile. Figure 4(b) shows the results of applying this analysis, which produces a residual error with a standard deviation of 1%. It is interesting to observe the difference in the character of the residual errors produced using the two approaches shown in Figs. 4(a) and 4(b). When Algorithm 1 is used, the residual error is “smoother” compared to when Eq. (11) is used, which results from the interpolants used in constructing A in Eq. (4). The overall magnitude and trends in the residual errors for the two approaches are about the same, implying that either approach is applicable to diffraction patterns without overlapping peaks, such as those shown in Fig. 3(a). In fact, due to its computational simplicity, analyzing such data with Eq. (11) might be preferred.

**FIG. 5.**
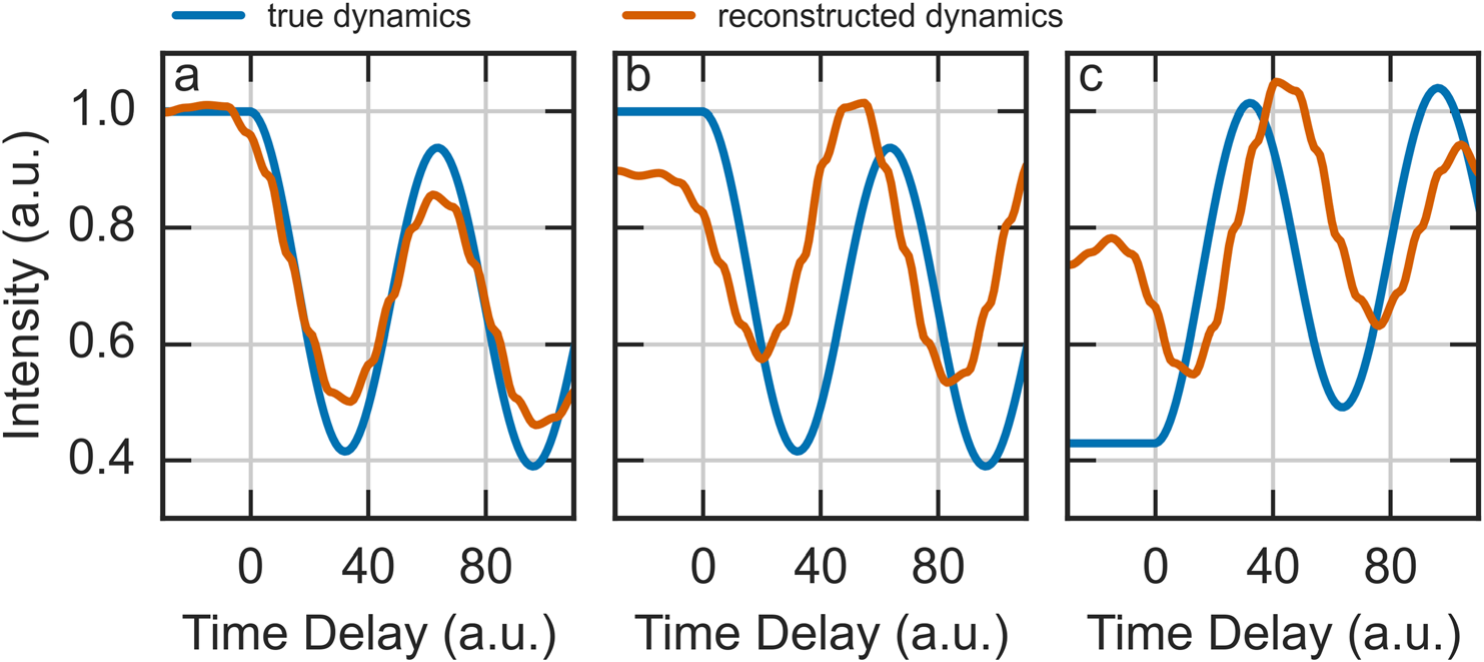
Comparison of the time-dependent intensity of the recovered diffraction data shown in Figs. 3(b) and 3(c). Shown for reference is the true amplitude that was used to generate the data. Panel (a) corresponds to the diffraction ring and (b) and (c) to the left and right spots in the overlapping spots, respectively.

The analysis technique presented here does a better job of matching the true dynamics than the traditional approach. In particular, in the case of a single diffraction spot, it successfully and accurately recovers the true dynamics. However, with more complicated diffraction, the quality of the reconstruction is worse, as is evident from Fig. 5(a), which shows the average intensity of the diffraction ring shown in Fig. 3(c) as a function of time-delay. Although the generate trends of the diffraction ring's amplitude are reproduced, the intensity of the oscillations is underestimated by about 20% at the peaks. This discrepancy is even more pronounced when considering the intensities of overlapping spots, which are shown in Figs. 5(b) and 5(c). Figure 5(b) plots the intensity of the left spot shown in Fig. 3(b), and Fig. 5(c) plots the intensity of the right spot. For both spots, the recovered amplitudes and phases of the oscillations are considerably different from the inputs used to simulate the data. The periods of the oscillations were accurately recovered. These discrepancies likely stem from the quality of the least-squares optimization, and it is expected that a more sophisticated optimization technique would result in better quantitative agreement. However, the traditional approach of analyzed streaked diffraction patterns cannot be applied to patterns containing overlapping spots or rings, and so, it is only through the use of a recovery algorithm based on Eq. (1) that the instantaneous diffraction patterns can be recovered. Furthermore, a simplification such as Eq. (11) cannot be made since the intensities of the various spots or rings are likely to vary independently; as such, an algorithm such as Algorithm 1 is the only viable way to analyze such data.

In the majority of simulations, it was observed that the recovered diffraction spots were broader and less intense than the true input diffraction spots. This is evident, for instance, by comparing the top and bottom rows in Fig. 3(a). It is unclear why this is the case; however, the average intensity which is plotted in Fig. 4(a) differs by less than a percent, and so, it appears that the overall intensity is conserved.

**FIG. 6.**
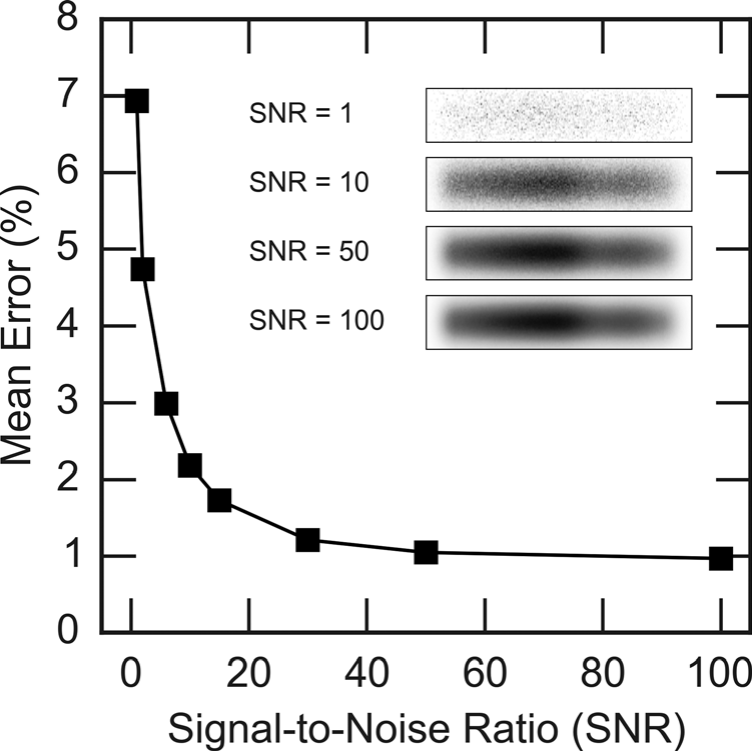
Percent error in the integrated diffraction intensity averaged over all time delays in the recovered diffraction patterns for various signal-to-noise ratios (SNRs). The inset shows examples of some of the simulated streaked diffraction spots with different SNR values.

The effectiveness of the reconstruction algorithm in the presence of significant noise contamination was then investigated. Using the dynamics of the Bragg peak shown in Fig. 3(a), various levels of noise were added prior to the reconstruction. For each SNR value, the input and recovered diffraction were compared by integrating the diffraction intensity over the Bragg peak and calculating the percent error at each time point. These values were then averaged over the entire time series to get the mean percent error. The results of this analysis are shown in Fig. 6.

As expected, the images with higher noise resulted in a worse reconstruction, due to the fact that the least squares solution is simply fitting to the noise. However, it should be noted that even in the presence of severe noise contamination, the reconstruction is quite robust, with the mean error remaining below 10%. This is likely the result of good initial guess for the underlying instantaneous diffraction pattern. This is an encouraging result that speaks about the feasibility of applying the analysis approach presented in this article to experimental data.

## VI. DISCUSSION

While these results have laid the foundation of the analysis of ultrafast streaked diffraction patterns, there are a few final remarks to be made: first, recall that the temporal resolution of a streaking experiment is $\sqrt{\Delta \tau_{\text{jitter}}^{2}+\Delta \tau_{\text{pump}}^{2}+\Delta \tau_{\text{camera}}^{2}}$, where Δ*τ*_jitter_ < 100 fs is the shot-to-shot timing jitter, Δ*τ*_pump_ ≈ 100 fs is the duration of the excitation pulse, and Δ*τ*_camera_ is the temporal resolution of the streak camera. Because the traditional analysis inherently couples the temporal and spatial coordinates, Δ*τ*_camera_ is the spatial resolution of the imaging system. This is typically taken to be the width of the unstreaked diffraction spot (divided by the streaking velocity *v_s_* to convert to time). Typical streak cameras have a resolution on the order of a few hundred femtoseconds,^26^ although recent advancements have crossed the 10 fs threshold,^30^ and so practically the overall temporal resolution of streaking experiments is limited by the quality of the streak camera.

However, this is no longer the case for streaked data that are analyzed by the method described in this paper. By completely decoupling the temporal and spatial coordinates, we were able to recover a diffraction image at every *W*/*L* pixel along the streaking direction. We therefore estimate the temporal resolution due to the recovery algorithm to be Δ*τ*_camera_ = *L*Δ*x*/*Wv_s_*, where Δ*x* is the pixel size along the streaking direction. Because there is a choice over the number *W* of interpolants used in the analysis, the fundamental temporal resolution limit of Δ*x*/*v_s_* ≈ 10 fs can be reached (when *W* =* L*). This comes at the cost of significantly increased computation time, and therefore, the presented analysis method is practically limited to a few hundred femtoseconds.

There are additionally some refinements that could improve the quality and speed of the reconstruction of the instantaneous diffraction patterns. For instance, instead of using fixed windows as the interpolants, it is possible to alternatively solve Eq. (5) for $\hat{u}$ and $\hat{\alpha }=\underset{\alpha ≽0}{\text{arg}\;\text{min}}{\left\|s-A(\alpha )\hat{u}\right\|}_{2}^{2}$, where $\alpha =[\alpha^{\left(0\right)};\alpha^{\left(1\right)};\ldots ;\alpha^{\left(W-1\right)}]$ is a vector formed by stacking the individual discrete interpolants and A(α) is given by Eq. (A6). This approach has been used in Ref. 42 to address a similar problem, and the authors found that it significantly improved the quality of the reconstruction by reducing the root-mean squared error by a factor of 4.

Another possible avenue to explore would be an implementation that exploits the parallel architecture of multithreaded Central Processing Units (CPUs) or Graphical Processing Units (GPUs). As this would dramatically reduce the computation time (since each row in the image could be analyzed in parallel instead of serially), the number *W* of interpolants could be larger, producing a better quality reconstruction.

Because of the relatively simple dynamics considered here, this article took a rudimentary approach to solving the inverse problem in Eq. (4) (that is, by using the least-squared solution). However, as evident in Fig. 5, this optimization approach is limited in its ability to recover quantitative amplitudes from complex diffraction patterns such as those containing rings or overlapping spots. In fact, for more complex diffraction patterns, such as those arising from organic crystals, a more sophisticated optimization technique such as maximum entropy might be warranted.^43^ This highlights that more work is needed to improve the quality of the reconstruction of streaked diffraction patterns based on Eq. (1).

Finally, it is worth noting that the analysis of streaked, time-dependent diffraction is formally analogous to the de-blurring of photographs. As such, the results presented here could potentially benefit from borrowing the results developed in that field (see, as a selection, Refs. 44–49). For example, an approximation similar to the one employed in Eq. (A3) is among the most promising and popular approaches to address spatially varying blur in photographic images, and so, it is worthwhile to keep abreast of progress on that front.

## VII. CONCLUSIONS

This article introduced the mathematical framework necessary to analyze time-dependent diffraction measured using ultrafast streak cameras. Through consideration of the physics that underlie the streaking process of a time-dependent diffraction pattern, Eq. (1) was derived as the governing equation for streaked ultrafast diffraction. By incorporating elements of Bayesian analysis, a procedure was formulated to reconstruct the complete set of instantaneous diffraction patterns from a single streaked image. A number of practical considerations were accounted for, culminating Algorithm 1, a practical algorithm that can be used to analyze experimental streaked data. This algorithm was tested on a number of simulated datasets, with results indicating a strong qualitative agreement between the input and reconstructed datasets. Furthermore, it was shown that this procedure works on streaked diffraction patterns containing overlapping spots or diffraction rings, thus opening up the technique of ultrafast streaking to several new classes of samples of interest, including organic crystals and polycrystalline materials.

## Figures and Tables

**FIG. 7.**
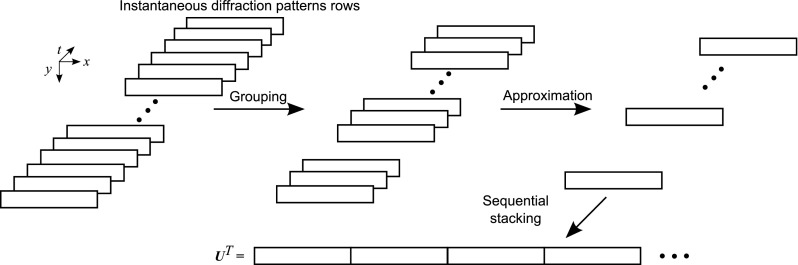
Illustration of the steps and approximations that lead to the formation of the vector U. First, the temporal coordinate is separated into *W* groups. Next, each group is approximated by a single diffraction pattern. Finally, the set of *W* vectors is sequentially stacked to form the vector U.

## APPENDIX: DERIVATION OF THE SPATIALLY VARYING OVERLAP-ADD METHOD

The main text derived Eq. (3) to relate the streaked diffraction pattern to the instantaneous diffraction patterns. This expression was calculated efficiently using the “spatially-varying overlap-add method.” To motivate this approximation, the time-dependent diffraction pattern will be re-written in the familiar “overlap-add” framework that is commonly used in the computation of discrete convolutions.^50^ The idea is to break the time-dependence of the image into discrete, overlapping “chunks” that are sliced from the full image stack by a set of *W* ≤* L* windowing functions ${\left\{\alpha^{\left(r\right)}(t)\right\}}_{r=0}^{W-1}$. In each temporal chunk, the diffraction pattern is treated as (temporally) constant. The windows are all of the same length but are only non-zero in a small temporal range. In the continuous representation, windowing *u*(*x*; *t*) takes the form

$$u(x;t)=\sum_{r=0}^{W-1}u(x;t)\alpha_{r}(t-rT),$$ (A1)

where *T* is the so-called “hop size,” which is the temporal spacing between adjacent windows. Equality is ensured by enforcing the windows to satisfy the following condition

$$\sum_{r=0}^{W-1}\alpha_{r}(t-rT)=1.$$ (A2)

The approximation now comes in the form of replacing each *u*(*x*; *t*) on the right-hand side of Eq. (A1) with a constant value in time; this constant value is chosen as the value of *u*(*x*; *t*) at the center of the temporal window so that $u(x;t)\alpha_{r}(t-rT)\approx u(x;rT)\alpha_{r}(t-rT)$. That is,

$$u(x;t)≃\sum_{r=0}^{W-1}u(x;rT)\alpha_{r}(t-rT),$$ (A3)

where equality holds when *W* =* L*. In this way, the windowing functions act as interpolants between the set of discrete points ${\left\{u(x;rT)\right\}}_{r=0}^{W-1}$, which approximate the continuous function *u*(*x*; *t*). These points are still continuous in their spatial coordinate and in the discrete representation will be represented as a set of vectors ${\left\{u^{\left(r\right)}\right\}}_{r=0}^{W-1}$. This allows for the space-variant convolution to be reduced to a linear combination of regular convolutions. With this approximation, Eq. (3) becomes

$$s_{j}=\sum_{k=0}^{L-1}\sum_{r=0}^{W-1}\rho_{k}\alpha_{k}^{\left(r\right)}u_{j-k}^{\left(r\right)}+n_{j},$$ (A4)

where the discrete versions of the interpolants are of the same length as the pulse profile; that is, $\alpha^{\left(r\right)}\in {\mathbb{R}}^{L\times 1}$ for *r* = 0,…,*W* − 1. If a vector U is constructed by sequentially stacking the vectors $u^{\left(r\right)}$ (see Fig. 7), then the above expression is linear in U and can be written succinctly as

s=AU+n, (A5)

where A is a matrix representing the convolution with the product of the discrete temporal pulse profile ρ and the discrete interpolants ${\left\{\alpha^{\left(r\right)}(t)\right\}}_{r=0}^{W-1}$. The matrix A can be efficiently calculated in the Fourier domain by modifying the so-called “efficient filter flow” method^32^

$$A=\sum_{r=0}^{W-1}I_{Q,Q+1}F^{H}\text{diag}\left(FZ_{\rho }\text{diag}(\alpha_{r})\rho \right)FZ_{u}P_{r}.$$ (A6)

There are many terms in this expression, defined as follows:

• $I_{m,n}$ is an *m* × *n* matrix with “1”s along its diagonal,
• F and $F^{H}$ are the discrete Fourier transform matrix and the discrete inverse Fourier transform matrix, respectively,
• $Z_{\rho }$ is a matrix that appends zeros to ρ such that its length equals *Q*,
• $Z_{u}$ is a matrix that appends zeros to the vector $u^{\left(r\right)}$ such that its length equals *Q*,
• $P_{r}$ is a matrix that chops $u^{\left(r\right)}$ from u,
• diag(x) is a square matrix with the vector x along its diagonal.

The computation of Eq. (A5) with A expressed as in Eq. (A6) can be performed quite quickly, on the order of $O(MW\;{\text{log}}_{2}MW)$ (Ref. 32), which is significantly faster than the naïve implementation in Eq. (3) when *W* ≪* L*.
